# Post-operative pain after root canal filling with two different obturation techniques: A prospective study

**DOI:** 10.4317/jced.62188

**Published:** 2024-10-01

**Authors:** Ana Ruiz-Cano, Irene Sánchez-Blanco, Juan J. Saúco-Márquez, Daniel Cabanillas-Balsera, Jenifer Martín-González, Juan J. Segura-Egea

**Affiliations:** 1 Department of Stomatology, Endodontics Section, School of Dentistry, University of Sevilla, Sevilla, Spain

## Abstract

**Background:**

Post-operative pain (POP) is a major problem in endodontics. This prospective study aims to investigate POP and the rate of analgesic intake after root canal treatment (RCT) using two obturation techniques: warm vertical compaction with epoxy resin-based sealer (WVT) versus sealer-based obturation (SBO) using a single-cone gutta-percha with calcium silicate-based sealer.

**Material and Methods:**

All RCT were carried out by the same endodontist, following a standardized protocol, and using two different obturation techniques: WVT with AH Plus sealer, and SBO with single-cone gutta-percha and AH Plus Bioceramic sealer. Patients finally assessed were 29 in the SBO group and 34 in the WVT group. After RCT, patients completed a 10-cm visual analogue scale (VAS) that ranked the level of pain. Results were analysed statistically using the Chi-square, ANOVA tests and logistic regression analysis.

**Results:**

Sex, age, number of roots, pulp diagnosis, periapical status, previous intake of NSAIDs or antibiotics did not affected POP. In the WVT group, 38.2% of patients felt some pain 24 hours post-treatment, while in the SBO group this percentage was 20.7% (*p* = 0.07). There were no significant differences in the need for analgesics in the week following treatment.

**Conclusions:**

The obturation technique used did not affect significantly the POP of patients after RCT. Although the percentage of patients in the SOB group showing POP was lower, there were no significant differences in the need to take analgesics.

** Key words:**Calcium silicate sealer, endodontic pain, post-operative pain, resin based sealer, sealer based obturation, single cone obturation, warm vertical compaction.

## Introduction

Endodontic patients associate fear of pain with the procedure itself, not with the post-treatment period (1). Certainly, pain control during endodontic treatment is always a challenge, but it can be achieved by properly using anesthetic techniques during operative procedures (2). However, post-operative pain, described as discomfort or pain experienced by the patient following root canal treatment (3), is distressing for both the patient and the operator (2). Post-operative pain, has been widely investigated (2-5). The intensity of POP is variable, ranging from mild discomfort to severe pain, and can occur just after RCT or up to several days later (6). POP occurs between 0% and 58% of RCT (7,8).

Mechanical factors, including working length determination (9), root canal instrumentation techniques (2,10,11), and over-instrumentation or extrusion of root-filling material have been associated to the presence of post-operative pain (12). However, the results of the studies that have investigated the influence of the obturation technique on postoperative pain are contradictory. Several studies have found that obturation technique influences POP (4,13,14), but others have reported no effect of root-filling technique (2,3,5,15). Regarding the effect of the sealer used on the postoperative pain, most of the studies have found no influence of the sealer on POP (12,16). Moreover, some studies have compared the POP felt by patients after root filling with the same obturation technique, but using different sealers, showing that the type of sealer did not influence postoperative endodontic pain (17).

In the last decade, calcium silicate based sealers (CSBS) have been incorporated to daily clinical endodontic practice as sealers in RCT. Premixed CSBS have excellent physicochemical and biological properties, and shown to be less cytotoxic compared to epoxy resin-based sealers (18). Therefore, root canal obturation with a premixed CSBS and a single gutta-percha cone (sealer-based obturation) (SBO) is now widely accepted (3,5).

Several studies have investigated the possible influence of CSBS on POP. A systematic review concluded that CSBS reduced POP after 24 h compared to a resin-based sealer (15). On the contrary, other clinical studies have found no significant differences in POP when comparing SBO and warm vertical compaction technique (WVT) with a resin-based sealer (3,19).

Until now, very few studies have been published comparing the SBO technique with CSBS and WVT with acrylic resin cement with respect to POP. One of the studies compares POP after both obturation techniques in vital teeth (20), and two others have evaluated POP after obturation with both techniques both in vital and necrotic teeth (3,5).

This prospective study aimed to investigate post-operative pain and the rate of analgesic intake after RCT using two obturation techniques: WVT compaction with epoxy resin-based sealer (AH Plus) versus SBO technique using a single-cone with a calcium silicate-based sealer (AH Plus Bioceramic).

## Material and Methods

This prospective clinical study has been conducted according to PROBE 2023 guidelines for reporting observational studies in Endodontics (21).

-Ethical approval

The study has been independently reviewed and approved by the Ethic Committee of the University of Sevilla, Sevilla, Spain (protocol number 0483). The study was undertaken with the understanding and written consent of each subject, and was conducted in full accordance with ethical principles, including the World Medical Association Declaration of Helsinki. All patients included in the study given their informed consent so that data from their medical history could be used in research.

-Study design and population

The prospective study was carried out in a private dental clinic in Andalusia, Spain. The subjects were recruited for the study from November 2022 to December 2023. The same dentist, specialized in Endodontics (Master in Endodontics) with 6 years of clinical experience (A.R-C.), carried out anamnesis, clinical examination, diagnosis, and root canal treatments in all patients. Patients needing RCT that met the inclusion/exclusion criteria were filled with either WVT using gutta percha with epoxy resin-based sealer (AH Plus, Dentsply Maillefer, York, PA, USA) or SBO technique using gutta percha single-cone with premixed calcium silicate-based sealer (AH Plus Bioceramic, Dentsply Maillefer, York, PA, USA). The treatment groups were assigned to an experimental group by alternating months.

-Inclusion and exclusion criteria

The inclusion criteria were as follows: Age over 18 years; No known allergies to any materials used in this study; RCT of a mature teeth with a probing pocket depth and mobility within normal limits was to be performed; The tooth had to be restorable; The patient signed the informed consent.

The exclusion criteria were as follows: Pregnancy or current breastfeeding; Root resorption; Immature teeth; History of previous RCT; History of cracked tooth syndrome or trauma; The tooth required a post; The patient does not sign the informed consent.

-Sample size calculation

The minimum sample size was determined using the calculation software of the National Center for Advancing Translational Sciences (NIH, UK) (National Center for Advancing Translational Sciences, NIH, UK, 2019). The sample size calculation model was used to compare two proportions with dichotomous results (https://sample-size.net/sample-size-proportions/). A significance level of α = 0.05 (two-tailed, 95% confidence level) was considered, an error β = 0.20 (statistical power of 80%), a prevalence of postoperative pain of 10% in a control group and 30% in the experimental group. The minimum sample size calculated was 62 patients.

-Root canal treatment protocol

The same operator, an endodontist with 6 years of experience, carried out all RCT following the same protocol, ensuring standardization. Prior to RCT, a systematic clinical and radiological examination were carried out. Patient sex and age, affected tooth, preoperative pain, pulpal and periapical diagnosis according to AAE (22), periapical status according to Periapical Index (23), and previous NSAID or antibiotic (AB) treatment were recorded.

The tooth was anaesthetized with Articaine 4% with 1:100,000 Epinephrine or, in hypertensive patients (20.0%), Mepivacaine 3% without vasoconstrictor, the volume of anaesthetic and type of injection being at the discretion of the dentist. Following an adequate anaesthesia and isolation with rubber dam, an endodontic access cavity was established. The working length was estimated using an apex locator (Dentaport ZX, Morita, Tokyo, Japan). Root canals were cleaned and shaped using crown-down technique. Protaper Gold rotary instruments (Dentsply-Sirona, Ballaigues, Switzerland), with torque control (X-Smart Plus), were used. As irrigating solution, 5.25% sodium hypochlorite (NaOCl) and 17% ethylenediaminetetraacetic acid (EDTA) were used. A minimum of 8 mL of NaOCl per canal was used and a semi-final rinse of 17% EDTA, with a minimum of 1 mL of EDTA per canal followed by a final rinse of 5.25% NaOCl. After cleaning and shaping, canals were dried and obturated. If the treatment required multiple visits, calcium hydroxide (Ca(OH)2) (AH Temp™, Dentsply Sirona, Bensheim, Germany) was used as an intra-canal medicament for 10–14 days and the tooth was sealed with a temporary restoration (3M™ Cavit™, 3M, Saint Paul, MI, USA).

The root filling technique for SBO was performed according to the manufacturer’s instructions (AH Plus Bioceramic, Dentsply Sirona, Charlotte, NC). The tip of the sealing syringe was inserted into the canal no deeper than the coronal third. A small amount of AH Plus Bioceramic was dispensed into the root canal. A matched gutta-percha point (Autofit, 0.4-0.6 taper, Kerr) was coated with a thin layer of sealer and inserted into the canal. Finally, excess gutta-percha was burned off at the orifice level using a heating device (Alfa unit of the B&L SuperEndo device, Biotech, USA) and condensed with a plugger. The excess sealer was then removed with a moist cotton pellet.

Warm vertical compaction technique (WVT) was carried out using both Alfa and Beta units of the B&L SuperEndo device (Biotech, USA). A matched gutta-percha point (Autofit, 0.4-0.6 taper, Kerr) was coated with a thin layer of AH Plus root canal sealer (Dentsply DeTrey) and inserted into the canal. Down pack was performed with Alfa unit, leaving 5-6 mm of apical gutta-percha, and the canal was then backfilled with the Beta unit. At the orifice level, the warm gutta-percha was condensed with a plugger and any excess sealer was removed with a moist cotton pellet.

After RCT completion, a periapical radiograph was taken. The tooth was restored with an appropriate definitive restoration either on the same visit or, if time did not permit, within a maximum of 1 week after RCT. Adjustment of the occlusion was made so that the restoration did not cause traumatic apical periodontitis.

-Pain assessment

Patients received instruction on how to use a 10-cm visual analogue scale (VAS) (24) to assess pain. Each patient self-recorded his/her pain by ranking the level of pain experienced before, during, and post- treatment at 24 h, 48 h, 72 h, and one week. These scores were converted to a numerical value between 0 and 10 and to a verbal scale, as follows: none (0), mild (1-3), moderate (4-6), severe (7-9), unbearable (10).

As had been done before treatment, after undergoing RCT patients were immediately questioned in relation to their pain perception during the treatment (intraoperative pain). Likewise, they were instructed to record their level of pain 1, 2, 3 and 7 days following treatment. The data necessary for the proposed study were rigorously collected and noted in their medical records.

Statistical analysis

Raw data were entered into Excel® (Microsoft Corporation, Redmond, WA, USA). Frequency distributions and contingency Table analyses were used to describe and compare independent variables with patient-reported pain (Chi-square test and ANOVA). Experienced pain variables were analysed first as continuous variables and then were dichotomized into high or low categories according to the sample distribution and previous literature reports on VAS (2,4,13,15). Multivariate logistic regression modelling technique was used.

To analyze the influence of the different variables on postoperative pain, Fisher’s exact test with Lancaster’s mid-P adjustment (25) was used. The Haldane-Anscombe correction was used to calculate the odd ratio when any of the cells caused division by zero. The significance level was set at *p* < 0.05.

## Results

**Figure 1:**
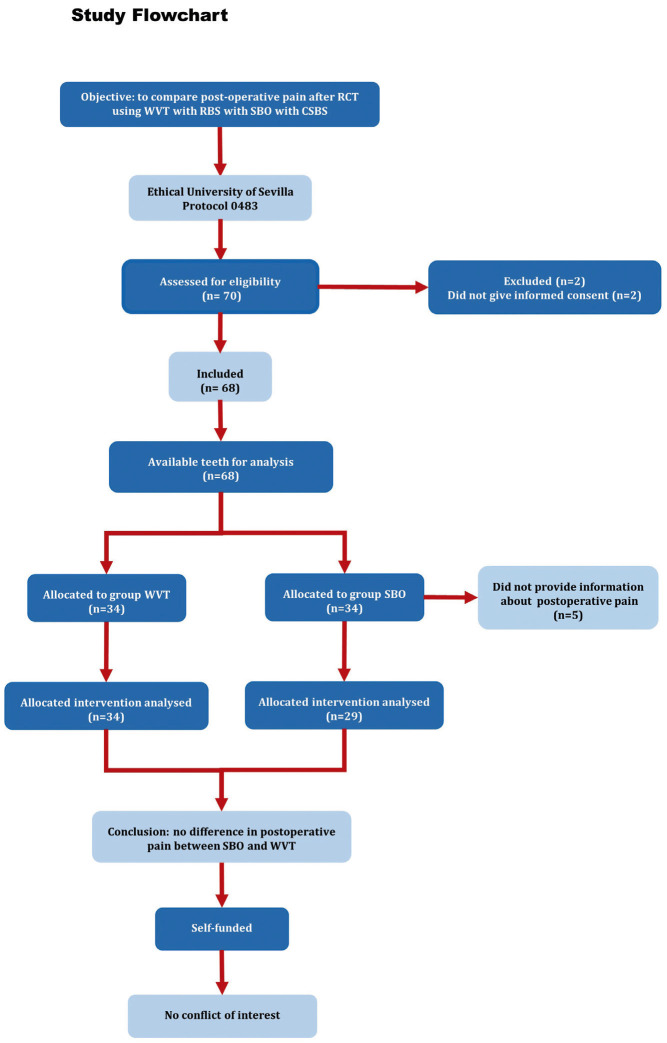
Study flowchart according to PROBE 2023 guidelines.

Initially, 70 patients, that met the inclusion criteria, were asked to participate in the study. However, two patients did not give their informed consent and were excluded. Then, 68 patients needing RCT were incorporated to the study (Fig. 1). Of them, 34 patients were included in the group treated with the SBO technique, and the remaining 34 were included in the group treated with WVT. However, five patients, all in the SBO group, although they have agreed to participate in the study, later did not provide information about POP. Therefore, the patients who could finally be assessed were 29 in the SBO group and 34 in the WVT group. The main demographic characteristics and initial clinical data of the patients in both treatment groups are shown in  Table 1.

There were no significant differences (*p* ≥ 0.05) between the two treatment groups in terms of age, sex, tooth type, pulp and periapical diagnosis, previous taking of analgesics or antibiotics, and preoperative pain.

Regarding intraoperative pain, patients in the SBO group felt pain during treatment at a significantly higher rate than those in the WVT group (OR = 4.8; 95% C.I. = 1.0 – 35.0; *p* = 0.048).

Concerning POP, in the first 24 hours after treatment, in the total sample nineteen patients (30.2%) felt some degree of pain ( Table 2). The median pain value was 0, with a range between 0 and 5. In the WVT group, 13 patients felt pain (38.2%), whereas in the SBO group only six patients (20.7%) felt pain, but there were no significant differences in POP between the two study groups (*p* = 0.07). The median pain value was 0 and the range was between 0 and 5 in both groups.

The percentage of patients feeling pain after RCT decreased over time in both groups, with no statistically significant differences observed between the two groups over the entire period (2, 3 and 7 days) analyzed (*p* ≥ 0.05) ( Table 2). Thus, no influence of the obturation technique on POP was observed (*p* > 0.05).

Seven patients (11.1%) required taking analgesics during the week following treatment, with no significant differences between the two groups (*p* > 0.05).

Regarding the level of pain ( Table 3), more than half of the patients had moderate or severe preoperative pain, without significant difference between groups (*p* > 0.05). Concerning intraoperative pain, 18 patients (28.9%) had moderate or severe pain. Although in the SBO group the percentage of patients who felt moderate or severe intraoperative pain was 37.9%, almost double than of the WVT group (20.6%), the difference was not significant (*p* > 0.05). The levels of postoperative pain felt by patients in both groups progressively decreased during the week after treatment, with no significant differences (*p* > 0.05).

## Discussion

The objective of the present study was to compare the POP felt by patients after endodontic treatment performed with two different obturation techniques: WVT compaction with epoxy resin-based sealer (AH Plus), and SBO technique using a single-cone with a calcium silicate-based sealer (AH Plus Bioceramic). Results showed that obturation technique used did not significantly affect the POP of patients after RCT. Results determine that the obturation technique used did not influence the need to use analgesics during the week following treatment.

A major problem with the POP study, and in general of any type of pain, is that the patient’s assessment is subjective. Therefore, the methodology used in the assessment of the level of pain is critical (26). In the present study, the standard methodology widely used in endodontics to assess POP has been used: pain was measured using the visual analogue scale, which is the standard validated method for pain assessment (24). Thus, the methodology used in this study is similar to that followed in other previously published studies (2,4,13,27). In addition, the pain has also been verbally analyzed for better understanding by the patients. The sample size was calculated so that the results could be significant.

All endodontists know the great importance of adequate pain control during treatment. Dental patients have become increasingly demanding about pain control during dental procedures (27). Therefore, in endodontics, offering the patient adequate local anesthesia is essential, and represents a practical development strategy that increases both patient loyalty and treatment acceptance (4).

Endodontic pain management should begin with preoperative pain control, which requires a correct diagnosis and control of the patient’s anxiety. Intraoperative pain control is also essential, for which very effective local anesthetics are available (2,27). Finally, postoperative pain management may involve a variety of pharmacological agents (5).

Minor complaints are relatively common after RCT (28), because in addition to pre-existing periapical inflammation in cases with apical periodontitis, minor mechanical and chemical trauma to the periapical tissues may also occur during treatment (26). In the present study, the operator, the same in all cases, always took special care to clean and shape the root canals, as well as to perform instrumentation and irrigation, with the greatest care to avoid causing periapical damage.

Another factor that may influence POP is the degree of cleaning and disinfection of the root canal system, as residual infection may lead to a painful exacerbation of the periapical inflammatory process (29). In the present study, in both study groups the disinfection protocol was the same and was performed by the same operator, so this factor probably did not influence POP. Furthermore, in both study groups there was a similar percentage of cases with symptomatic apical periodontitis and apical abscess.

Obturation technique is a main factor implicated in POP (2). Root filling always carries a risk of extrusion of the filling material, which is associated with POP (15). In the present study, patients in the SBO group felt pain during treatment (intraoperative pain) at a significantly higher rate than those in the WVT group. These findings are in agreement with previous results (4), who found higher pain levels associated to Thermafil obturation technique, probably explained by the extrusion of gutta-percha that frequently occurs when these techniques are used. Similar results have been reported by other investigators (30). However, other studies have not found correlation between sealer extrusion and post-obturation pain prevalence (2).

The results of the present study have found no influence of the obturation technique in the POP between the first and seventh day after treatment. POP levels following root canal therapy are affected by the obturation technique, especially first 4 days following obturation (11). However, coinciding with the results of this study, it has been reported that there were no differences in POP when comparing warm vertical compaction technique and single cone guttapercha (5), which are the two obturation techniques used in this study. Moreover, recently it has been that warm vertical compaction technique and single cone guttapercha are associated with similar POP levels and analgesics intake as WVC with resin-based sealer (3).

Concerning the cement sealer used, the results of studies that have investigated the possible relationship of type of cement used with the level of POP are inconclusive. Mekhdieva *et al*. (2021) (13) found that obturation with gutta-percha/bioceramic sealer was associated with significantly lower short-term POP, and with a trend for lower analgesic intake and flare-up incidence, as compared to gutta-percha/traditional sealer techniques. On the contrary, other studies have found no influence of the sealer in the POP (3,5,17).

The present study have limitations that should be taken into account when the results are interpreted. In a clinical investigation such as the one in this study, it is difficult to determine whether a single factor or several factors are responsible for POP. Since each technique used a different type of sealer, the influence of the type of sealer cement could be responsible for the differences in POP. Since each of the techniques must be performed with a specific type of sealer, a study comparing the WVT technique with AH Plus Bioceramic cement with the SOB technique with this same sealer cannot be designed. Definitively, it is not known whether the difference observed in POP between the two groups is due more to the type of root filling technique than to the type of sealer.

Conclussions

The obturation technique used did not affect significantly the POP of patients after RCT. Although the percentage of patients in the SOB group showing POP was lower, there were no significant differences in the need to take analgesics.

## Figures and Tables

**Table 1:** Baseline demographics and clinical features of participants included in the study.

Variable	WVT with AH Plus (n = 34)	SBO with AH Plus Bioceramic (n = 29)	Total Sample (n = 63)	p
Sex				
Men	18 (52.9%)	17 (58.6%)	35 (55.6%)	0.66
Women	16 (47.1%)	12 (41.4%)	28 (44.4%)	
Age	44.1 ±14.2	45.6 ±13.1	44.8 ±13.6	0.67
Tooth type				
One-rooted	6 (17.6%)	6 (20.7%)	12 (19.0%)	0.76
Multi-rooted	28 (82.4%)	23 (79.3%)	51 (81.0%)	
Pulp diagnosis				
Irreversible pulpitis	17 (50%)	14 (48.3%)	32 (50.8%)	0.89
Pulp necrosis	17 (50%)	15 (51.7%)	31 (49.2%)	
Periapical diagnosis				
Normal / Asymptomatic AP	24 (70.6%)	22 (75.9%)	46 (73.0%)	0.65
Symptomatic AP / Apical abscess	10 (29.4%)	7 (24.1%)	17 (27.0%)	
Previous AINE				
No	7 (20.6%)	9 (31.0%)	16 (25.4%)	0.36
Yes	27 (79.4%)	20 (69.0%)	47 (74.6%)	
Previous antibiotic intake				
No	28 (82.4%)	19 (65.5%)	47 (74.6%)	0.14
Yes	6 (17.6%)	10 (34.5%)	16 (25.4%)	
Preoperative pain intake				
No	4 (11.8%)	2 (6.9%)	6 (9.5%)	0.55
Yes	30 (88.2%)	27 (93.1%)	57 (90.5%)	
Median	3.5	4	4	
Range	0-7	0-8	0-8	
Intraoperative pain				
No	9 (26.5%)	2 (6.9%)	11 (17.5%)	0.048*
Yes	25 (73.5%)	27 (93.1%)	52 82.5%)	
Median	2	3	2	
Range	0-7	0-7	0-7	

WVT: Warm Vertical Compaction Technique SBO: Sealer Based Obturation Technique * *p*< 0.05

**Table 2:** Postoperative pain levels in both groups and in the total sample.

Variable	WVT with AH Plus (n = 34)	SBO with AH Plus Bioceramic (n = 29)	Total sample (n = 63)	p
Postoperative pain at 24 h	-			
No	21 (61.8%)	23 (79.3%)	44 (69.8%)	0.07
Yes	13 (38.2%)	6 (20.7%)	19 (30.2%)	
Median	0	0	0	
Range	0-5	0-5	0-5	
Postoperative pain at 48 h				
No	31 (91.2%)	26 (89.7%)	57 (90.5%)	0.42
Yes	3 (8.8%)	3 (10.3%)	6 (9.5%)	
Median	0	0	0	
Range	0-2	0-5	0-5	
Postoperative pain at 72 h				
No	31 (91.2%)	27 (93.1%)	58 (92.1%)	0.81
Yes	3 (8.8%)	2 (6.9%)	5 (7.9%)	
Median	0	0	0	
Range	0-1	0-3	0-3	
Postoperative pain at 7 days				
No	34 (100%)	29 (100%)	63 (100%)	0.46
Yes	0 (0%)	0	0	
Median	0	0	0	
Range	0	0	0	
Postoperative medication				
No	30 (88.2%)	26 (89.7%)	56 (88.9%)	0.88
Yes	4 (11.8%)	3 (10.3%)	7 (11.1%)	

WVT: Warm Vertical Compaction Technique SBO: Sealer Based Obturation Technique

**Table 3:** Number and percentage of patients in relation to the level of pain felt throughout the study in both groups.

Pain Level	Preop. pain		Intraop. pain		POP at 24 h		POP at 48 h		POP at 72 h		POP at 7 days	
	WVT	SBO	WVT	SBO	WVT	SBO	WVT	SBO	WVT	SBO	WVT	SBO
None	4 (11.7)	2 (6.9)	9 (26.5)	2 (6.9)	21 (61.8)	23 (79.3)	31 (91.2)	26 (89.7)	31 (91.2)	27 (93.1)	34 (100)	29 (100)
Mild	13 (38.2)	8 (27.6)	18 (52.9)	16 (55.2)	11 (32.4)	5 (17.2)	3 (8.8)	2 (6.9)	3 (8.8)	2 (6.9)	0	0
Moderate	11 (32.4)	13 (44.8)	5 (14.7)	9 (31.0)	2 (5.9)	1 (3.4)	0	1 (3.4)	0	0	0	0
Severe	6 (17.6)	6 (20.7)	2 (5.9)	2 (6.9)	0	0	0	0	0	0	0	0
Unbearable	0	0	0	0	0	0	0	0	0	0	0	0

WVT: Warm Vertical Compaction Technique group. SBO: Sealer Based Obturation Technique group. POP: Postoperative pain.

## Data Availability

The datasets used and/or analyzed during the current study are available from the corresponding author.
